# Supplementary material for: Role and mechanism of NCAPD3 in promoting malignant behaviors in gastric cancer
Source: Front Pharmacol. 2024 Apr 22;15:1341039. doi: 10.3389/fphar.2024.1341039 (PMC11070777; doi:10.3389/fphar.2024.1341039)
Supplement: Supplementary file 11 [file DataSheet2.ZIP › GSEA/Oncogenic signature/my_analysis.Gsea.1599461962066/EGFR_UP.V1_UP.html]

Details for gene set EGFR\_UP.V1\_UP[GSEA]

|  || Dataset | filtered\_dataset.sample\_info.cls#WT\_versus\_NCAPD3\_MUT |
| Phenotype | sample\_info.cls#WT\_versus\_NCAPD3\_MUT |
| Upregulated in class | NCAPD3\_MUT |
| GeneSet | EGFR\_UP.V1\_UP |
| Enrichment Score (ES) | -0.39958456 |
| Normalized Enrichment Score (NES) | -2.2149382 |
| Nominal p-value | 0.0023201855 |
| FDR q-value | 0.00863512 |
| FWER p-Value | 0.028 |
Table: GSEA Results Summary

  

Fig 1: Enrichment plot: EGFR\_UP.V1\_UP      
 Profile of the Running ES Score & Positions of GeneSet Members on the Rank Ordered List

  

| SYMBOL | TITLE | RANK IN GENE LIST | RANK METRIC SCORE | RUNNING ES | CORE ENRICHMENT || 1 | 10161 | LPAR6 | 163 | 0.711 | -0.0765 | No |
| 2 | 9456 | HOMER1 | 198 | 0.680 | -0.0612 | No |
| 3 | 2244 | FGB | 321 | 0.582 | -0.1155 | No |
| 4 | 6197 | RPS6KA3 | 643 | 0.403 | -0.3248 | No |
| 5 | 84159 | ARID5B | 699 | 0.379 | -0.3424 | No |
| 6 | 2114 | ETS2 | 703 | 0.375 | -0.3226 | No |
| 7 | 25825 | BACE2 | 756 | 0.344 | -0.3401 | No |
| 8 | 6558 | SLC12A2 | 839 | 0.276 | -0.3833 | Yes |
| 9 | 8835 | SOCS2 | 840 | 0.275 | -0.3672 | Yes |
| 10 | 6303 | SAT1 | 850 | -0.233 | -0.3600 | Yes |
| 11 | 3673 | ITGA2 | 858 | -0.264 | -0.3496 | Yes |
| 12 | 9582 | APOBEC3B | 868 | -0.278 | -0.3398 | Yes |
| 13 | 2150 | F2RL1 | 887 | -0.304 | -0.3350 | Yes |
| 14 | 4680 | CEACAM6 | 955 | -0.374 | -0.3616 | Yes |
| 15 | 9314 | KLF4 | 972 | -0.389 | -0.3504 | Yes |
| 16 | 6286 | S100P | 1011 | -0.412 | -0.3537 | Yes |
| 17 | 11138 | TBC1D8 | 1026 | -0.424 | -0.3390 | Yes |
| 18 | 9076 | CLDN1 | 1084 | -0.463 | -0.3531 | Yes |
| 19 | 3669 | ISG20 | 1097 | -0.475 | -0.3339 | Yes |
| 20 | 8727 | CTNNAL1 | 1106 | -0.482 | -0.3114 | Yes |
| 21 | 5277 | PIGA | 1126 | -0.500 | -0.2958 | Yes |
| 22 | 3887 | KRT81 | 1168 | -0.536 | -0.2940 | Yes |
| 23 | 2119 | ETV5 | 1176 | -0.545 | -0.2671 | Yes |
| 24 | 6310 | ATXN1 | 1177 | -0.547 | -0.2349 | Yes |
| 25 | 64759 | TNS3 | 1197 | -0.572 | -0.2151 | Yes |
| 26 | 23657 | SLC7A11 | 1205 | -0.581 | -0.1860 | Yes |
| 27 | 10150 | MBNL2 | 1217 | -0.591 | -0.1592 | Yes |
| 28 | 22822 | PHLDA1 | 1255 | -0.643 | -0.1483 | Yes |
| 29 | 1956 | EGFR | 1266 | -0.657 | -0.1169 | Yes |
| 30 | 1839 | HBEGF | 1328 | -0.751 | -0.1170 | Yes |
| 31 | 2650 | GCNT1 | 1342 | -0.781 | -0.0806 | Yes |
| 32 | 22936 | ELL2 | 1370 | -0.882 | -0.0483 | Yes |
| 33 | 4312 | MMP1 | 1407 | -1.304 | 0.0022 | Yes |
Table: GSEA details [plain text format]

  

Fig 2: EGFR\_UP.V1\_UP      
 Blue-Pink O' Gram in the Space of the Analyzed GeneSet

  

Fig 3: EGFR\_UP.V1\_UP: Random ES distribution      
 Gene set null distribution of ES for **EGFR\_UP.V1\_UP**
